# Antibody-mediated biorecognition of myelin oligodendrocyte glycoprotein: computational evidence of demyelination-related epitopes

**DOI:** 10.1038/s41598-018-36578-8

**Published:** 2019-02-14

**Authors:** Jéssica Cristiane Magalhães Ierich, Doralina Guimarães Brum, Ariana de Souza Moraes, Akemi Martins Higa, Pâmela Soto Garcia, Celina Massumi Miyazaki, Marystela Ferreira, Luís Antonio Peroni, Guedmiller Souza de Oliveira, Eduardo de Faria Franca, Luiz Carlos Gomide Freitas, Fabio Lima Leite

**Affiliations:** 1 Nanoneurobiophysics Research Group, Department of Physics, Chemistry and Mathematics, Federal University of São Carlos, Sorocaba, 18052-780 Brazil; 2 Institute of Tropical Medicine of São Paulo, University of São Paulo, São Paulo, 05403-000 Brazil; 3 Department of Neurology, Psychology and Psychiatry, UNESP - São Paulo State University, Botucatu, 18618-687 Brazil; 4 Science and Technology Centre for Sustainability, Federal University of São Carlos, Sorocaba, 18052-780 Brazil; 5 Rheabiotech Laboratory Research and Development, Campinas, 13084-791 Brazil; 6 Institute of Chemistry, Federal University of Uberlândia, Uberlândia, 38400-902 Brazil; 7 Department of Chemistry, Federal University of São Carlos, São Carlos, 13565-905 Brazil

## Abstract

Antigen-antibody interaction is crucial in autoimmune disease pathogenesis, as multiple sclerosis and neuromyelitis optica. Given that, autoantibodies are essential biomolecules, of which the myelin oligodendrocyte glycoprotein (MOG) can figure as a target. Here we combined Molecular Dynamics (MD), Steered Molecular Dynamics (SMD), and Atomic Force Microscope (AFM) to detail MOG recognition by its specific antibody. The complex model consisted of the MOG external domain interacting with an experimental anti-MOG antibody from the Protein Data Bank (1PKQ). Computational data demonstrated thirteen MOG residues with a robust contribution to the antigen-antibody interaction. Comprising five of the thirteen anchor residues (ASP_102_, HIS_103_, SER_104_, TYR_105_, and GLN_106_), the well-known MOG_92–106_ peptide in complex with the anti-MOG was analysed by AFM and SMD. These analyses evidenced similar force values of 780 pN and 765 pN for computational and experimental MOG_92–106_ and anti-MOG detachment, respectively. MOG_92–106_ was responsible for 75% of the total force measured between MOG external domain and anti-MOG, holding the interaction with the antibody. The antigen-antibody binding was confirmed by Surface Plasmon Resonance (SPR) measurements. Combined approaches presented here can conveniently be adjusted to detail novel molecules in diseases research. This can optimize pre-clinical steps, guiding experiments, reducing costs, and animal model usage.

## Introduction

Mechanisms related to healthy and pathogenic events in organisms depend on processes of biorecognition and interaction, particularly those involved in immune response as antigen-antibody binding^1^. Antibodies are highly-specialized proteins that recognize structural and chemical patterns of foreign elements, named antigens. An antigen-antibody interaction presents specificity and high affinity determined by the complementarity-determinant region (CDR), which is formed by six variable loops in the light (L1, L2, and L3) and heavy (H1, H2, and H3) chains of the antibody^1–3^. In light of their features during an autoimmune response, antibodies are shown to be important by targeting endogenous components in the pathogenesis of demyelinating diseases as multiple sclerosis (MS) and neuromyelitis optica spectrum disorders (NMOSD)^4^.

In this context, the myelin oligodendrocyte glycoprotein (MOG) has been extensively investigated as a target of autoantibodies in demyelinating diseases’ mechanism^5,6^, especially in MS^7,8^ and NMOSD^9,10^. MOG is a protein with 28 kDa expressed only in the central nervous system (CNS)^11^. This protein is found in oligodendrocytes and myelin sheath of CNS neurons, representing about 0.05% of the total myelin protein^11^. The function of MOG remains unclear, but its late expression in the CNS suggests an involvement in the compaction and maintenance of the myelin structure^5^. Significant information about MOG in the CNS immune response came from experimental autoimmune encephalomyelitis (EAE), an important animal model in demyelinating diseases investigation^12^. Currently, available data suggest that antibodies against MOG are not restricted to a disease in particular, but could indicate the demyelination of the CNS^5,13^. In spite of all obtained data, new approaches are needed to complement and enhance available data on the correlation between MOG and demyelinating diseases^9,11,14^.

Considering the rapid development of nanoscience and nanotechnology, advanced computational methods could be valuable tools for biomolecular interaction description and comprehension as well as they can extensively contribute to the understanding of MOG as a target during the demyelination process^15^. The application of computational techniques of modelling and simulation in the demyelinating disease research is in the beginning, but showed promising results in the description and characterisation of autoantigens, antigen presenting process, and T-cell activation^16,17^.

In this work, computational approaches were implemented in the MOG-antibody 3D complex, considering MOG external domain and MOG immunogenic peptides, aiming structural and dynamic data generation for demyelinating diseases understanding. Here, the MOG-antibody interaction was simulated by means of Molecular Dynamics (MD), together with Steered Molecular Dynamics (SMD) and Atomic Force Microscopy techniques, which have identified residues in the MOG structure that anchored the antigen-antibody complex and demonstrated a huge contribution of the MOG_92–106_ encephalitogenic peptide holding the interaction between the specific external domain of MOG and an experimental anti-MOG antibody.

## Results

### Antigen-antibody structural fluctuation during complex formation

**Figure 1:**
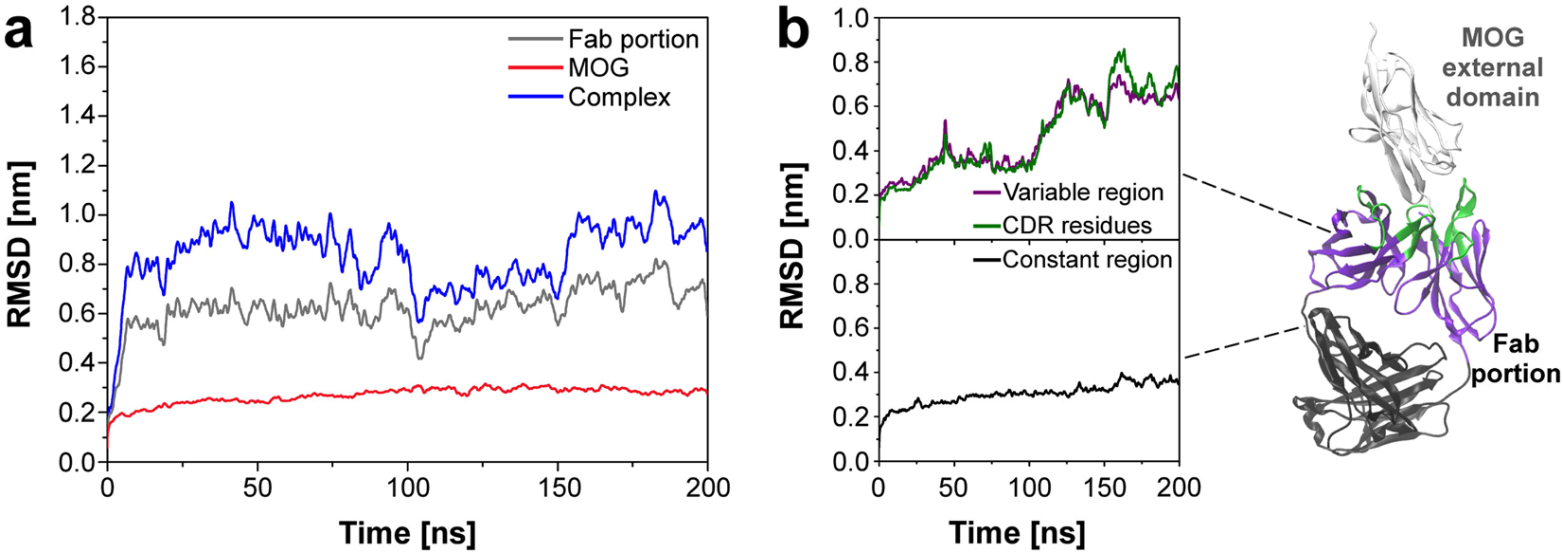
Evolution of system biomolecules RMSD values during the simulation time from the initial structure. **(a)** Complex, Fab, and MOG RMSD values calculated during the entire simulation. **(b)** RMSD evolution of each Fab region as follows: variable region, CDR, and constant region.

In order to detail the dynamics of the antigen-antibody interaction, the complex formed by MOG external domain and Fab portion of the experimental MOG-specific antibody, previously described by Breithaupt *et al*.^6^, was simulated using MD programs for 200 ns. The structural variation of both MOG and demyelinating antibody Fab portion was monitored and evaluated concerning root-mean-square deviation (RMSD) calculation. RMSD values were obtained considering (a) the anti-MOG Fab portion only; (b) MOG external domain only; and (c) the complex composed of MOG and anti-MOG Fab molecules. Fig. 1a highlights a difference in the structural variation pattern between anti-MOG Fab and MOG external domain. The anti-MOG Fab molecule showed a larger conformational fluctuation than MOG protein during the simulation, presenting average values of 0.63 ± 0.08 nm against 0.30 ± 0.03 nm, respectively. Additionally, Fig. 1b explores the contributions of each Fab region, in which the variable region, including CDR residues, fluctuated more than the constant region. CDR loops, composed of 55 residues in the variable region, were identified in the anti-MOG Fab structure using the abYsis system^18^ as highlighted in green in the complex structure (Fig. 1b).

### Biomolecules structure and antigen-antibody interaction maintenance

The formation of hydrogen bonds between the complex biomolecules and water as well as salt bridges was monitored in the course of MD simulation. Table 1 compares both values obtained in every 20 ns of simulation, which shows that the number of salt bridges decreased, and the hydrogen bond formation increased along the simulation time. Average values calculated for hydrogen bonds and salt bridge formation were 2,093 ± 56 and 65 ± 4, respectively.

**Table 1:** Number of hydrogen bonds between water molecules and proteins as well as salt bridges quantified during the simulation.

Interaction	20 ns	40 ns	60 ns	80 ns	100 ns	120 ns	140 ns	160 ns	180 ns	200 ns
Hydrogen bonds	1,894	2,071	2,107	2,113	2,142	2,116	2,120	2,149	2,194	2,181
Salt bridges	72	69	63	64	63	63	65	63	65	62

**Figure 2:**
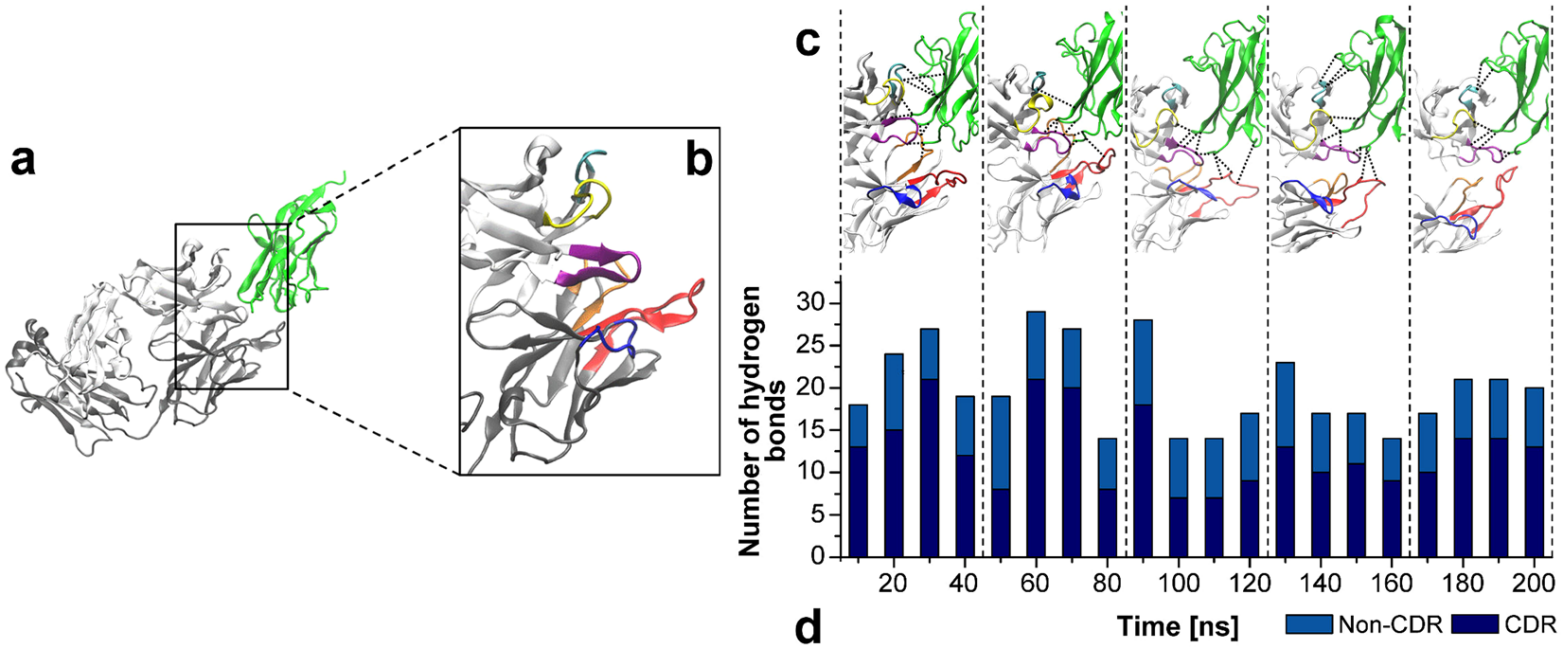
Hydrogen bond contribution for antigen-antibody complex formation. **(a)** The complex formed by the MOG external domain (green) and the anti-MOG Fab portion (light chain highlighted in white and heavy chain in grey). **(b)** Identification of the six CDR in the anti-MOG Fab structure, in which L1 is shown in red, L2 in blue, L3 in orange, H1 in yellow, H2 in cyan, and H3 in purple. **(c)** Contributions of CDR residues in terms of hydrogen bonds (black dashed lines) in the interaction between MOG (green) and anti-MOG Fab for every 40 ns of simulation. **(d)** Hydrogen bonds quantified for the complex in every 10 ns of simulation.

Also, considering hydrogen bonds are crucial for the antigen recognition, and binding by an antibody molecule^19^, they were quantified during the simulation. These measurements considered hydrogen bonds formed between (a) anti-MOG Fab residues and MOG external domain, and (b) CDR residues and MOG external domain (Fig. 2). All the analyses were based on CDR information in the anti-MOG structure described previously using the abYsis system (Fig. 2a,b). The obtained data highlight a CDR contribution of 60% in the total hydrogen bonds involved in the complex maintenance along the simulation (Fig. 2d). In this context, the anti-MOG Fab heavy chain contributed more for antigen-antibody binding than the light chain. Among the six CDRs, H3, H2, and L3 were the most interactive against a minor support from H1, L1, and L2 (Fig. 2c).

Concerning residues in the MOG external domain structure that anchor the complex with the anti-MOG Fab portion (Fig. 2c); they were identified and ranked according to hydrogen bonds contribution. Thus, from the highest to the lowest contributor, the thirteen MOG residues involved in antigen-antibody hydrogen bonds were: GLU_108_, GLY_1_, SER_104_, HIS_103_, ASN_53_, THR_33_, ASP_102_, GLN_106_, GLU_107_, ARG_52_, GLN_4_, TYR_105_, and TYR_40_.

### Complex free-binding energy analysis

**Figure 3:**
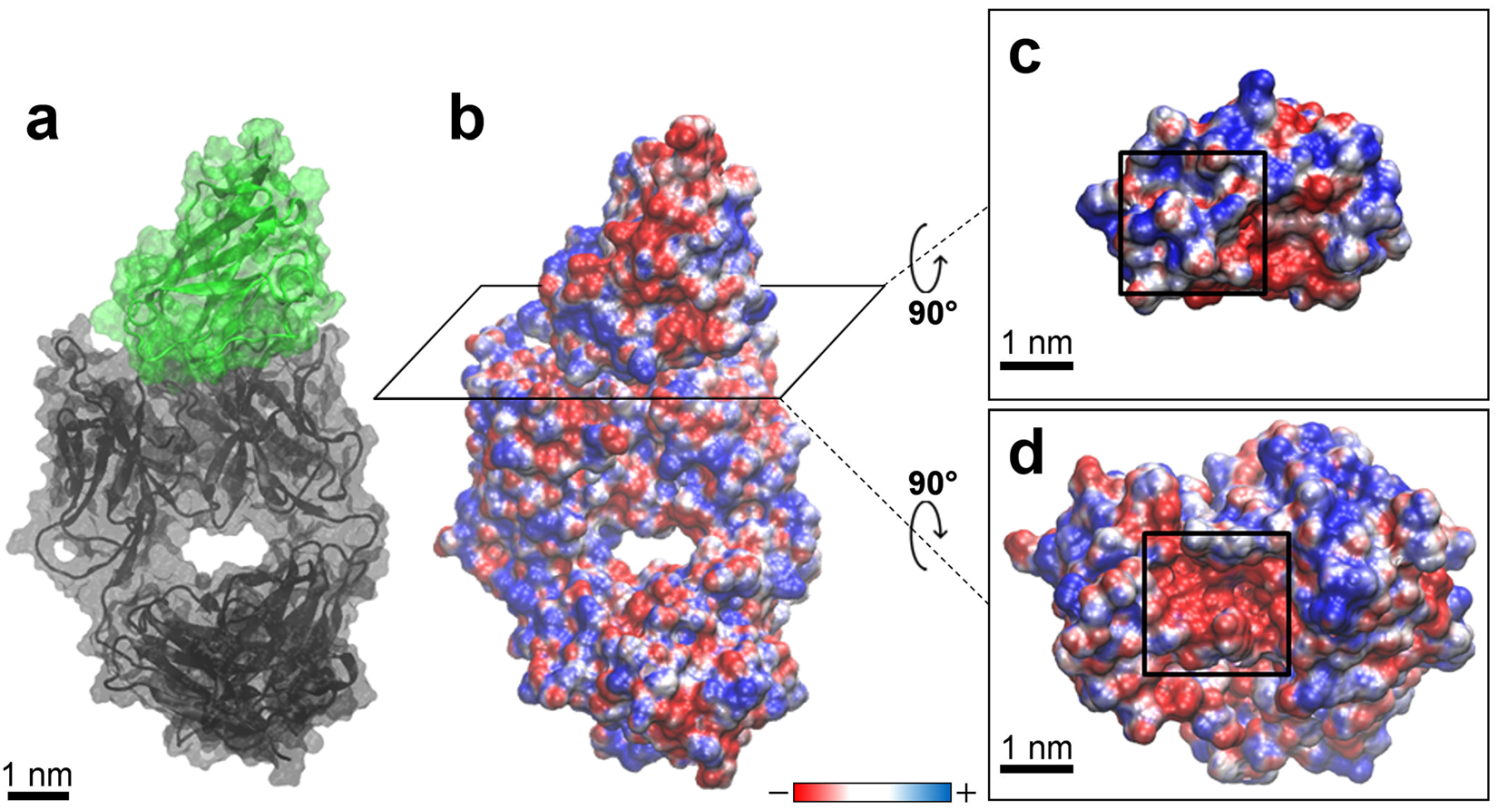
Electrostatic potential (−3.0 K_B_T/e to + 3.0 K_B_T/e) of the MOG-Fab complex. **(a)** The complex structure composed of MOG external domain (green) and the anti-MOG Fab portion (black). **(b)** Complex electrostatic potential representation in which the interaction site is represented by the horizontal plane. **(c)** Superior view of the horizontal plane with the most interactive region of the MOG molecule comprised in the dark square. **(d)** Inferior view of the horizontal plane with the most interactive region of the anti-MOG Fab portion comprised in the dark square.

The complex formed by MOG and anti-MOG Fab (Fig. 3a) was described in terms of free-binding energy (ΔG_bind_) considering parts of the MD trajectory with the lowest RMSD values. Based on the data presented in Fig. 1a, ΔG_bind_ calculation considered 15,000 frames, more precisely from 53 to 68 ns of the simulation, in which was observed a less structural fluctuation. Then, using an interval of 10 ps for the measurements, a set of 1,500 energy values was obtained from the MD trajectory. The average ΔG_bind_ calculated for the complex was −43.1 ± 17.6 kcal mol^−1^. Our data for electrostatic (ΔE_elec_) and van der Waals (ΔE_vdW_) contribution to the ΔG_bind_ calculation were, respectively, −438.4 ± 45.9 kcal mol^−1^ and −79.0 ± 6.4 kcal mol^−1^. This lower value for ΔE_elec_ in comparison with ΔE_vdW_ suggested a crucial electrostatic contribution to the complex formation and maintenance. Electrostatics features of the complex were analysed according to the electrostatic potential obtained by APBS (Adaptive Poisson-Boltzmann Solver)^20^ program (Fig. 3b). Binding sites of both MOG external domain and the anti-MOG Fab portion (Fig. 3c,d, respectively) showed the most interactive regions oppositely charged, indicating a significant role of electrostatics for antigen-antibody complex formation and maintenance.

### SMD and AFM forces involved in MOG-antibody binding

**Figure 4:**
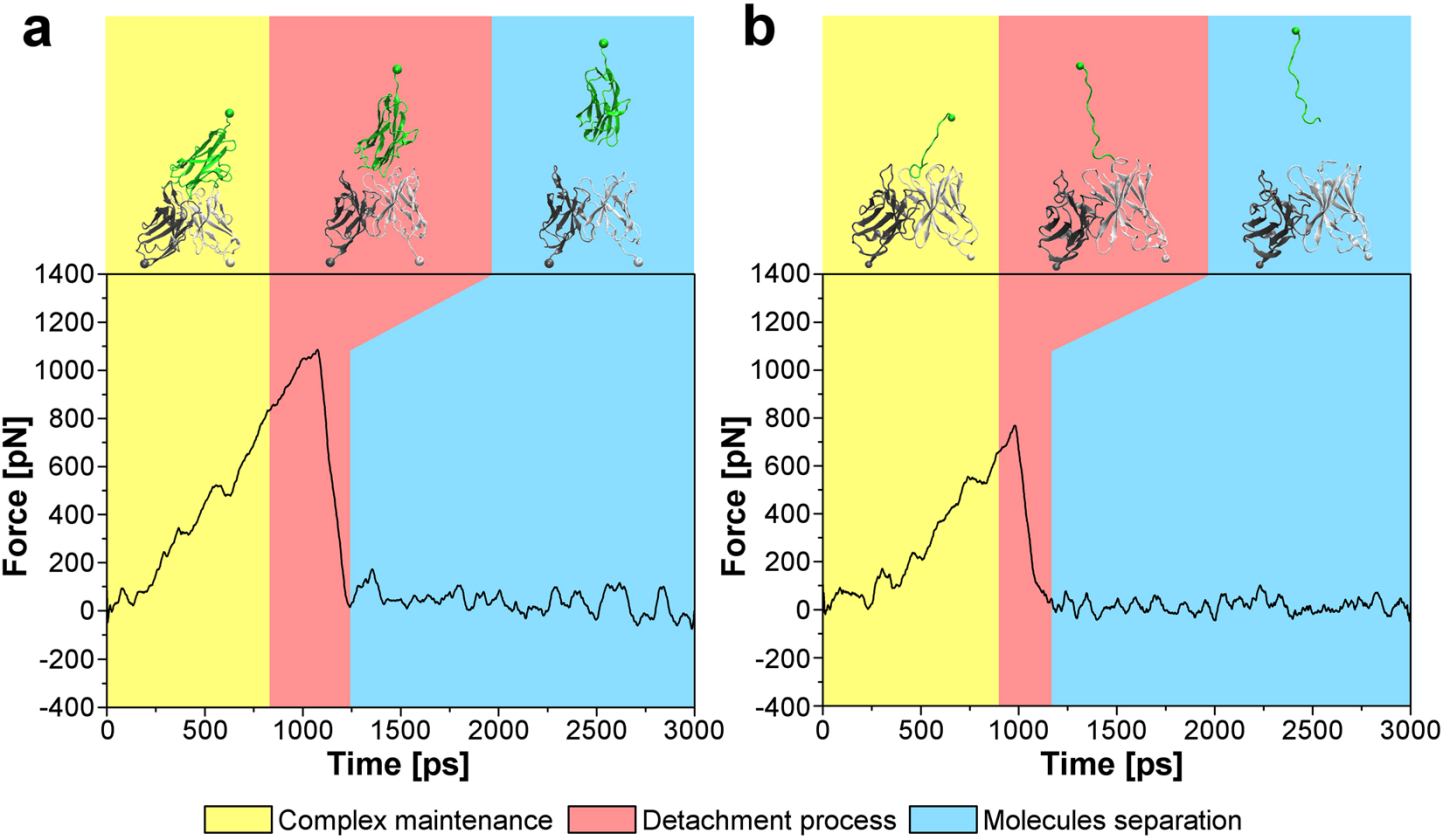
Computational forces involved in the antigen-antibody complex. **(a)** SMD force curve of the interaction between MOG external domain and the anti-MOG Fv portion. **(b)** SMD force curve of the interaction between the MOG_92–106_ peptide and the anti-MOG Fv portion.

Force values involved in the antigen-antibody interaction were obtained from a set of 40 SMD simulation data considering both the entire external domain of MOG and immunogenic MOG_92–106_ peptide. The MOG_92–106_ was chosen for the simulations considering the high proportion of the thirteen MOG anchor residues, identified by hydrogen bond analysis in Fig. 2, concentrated in this peptide (5 of the 13 residues were included in MOG_92–106_), as better explained in the Discussion section. Fig. 4a shows the evolution of the applied force during 3 ns of simulation considering MOG external domain and the variable region of the anti-MOG Fab portion (Fv) system. According to the presented data, the unbinding event occurred in about 1 ns of SMD simulation after the application of an external force of 1,042 pN, with a standard deviation of 192 pN among all the 20 simulations data.

Fig. 4b illustrates the unbinding dynamics of the complex formed by MOG_92–106_ immunogenic peptide and the anti-MOG Fv portion based on force values presented in the 20 simulations performed. The average unbinding force was 780 pN measured after about 970 ps of simulation, in which a standard deviation of 128 pN was observed. The Fig. 4 highlights the main steps of the SMD unbinding process: complex maintenance (yellow region), in which the antigen-antibody binding force is higher than the applied external force; detachment process (pink region), in which the applied force is high enough to detach MOG external domain from the Fv portion; and molecules separation (blue region), in which molecules have no interaction. Biomolecules structural changes are also represented in the upper panel of Fig. 4.

**Figure 5:**
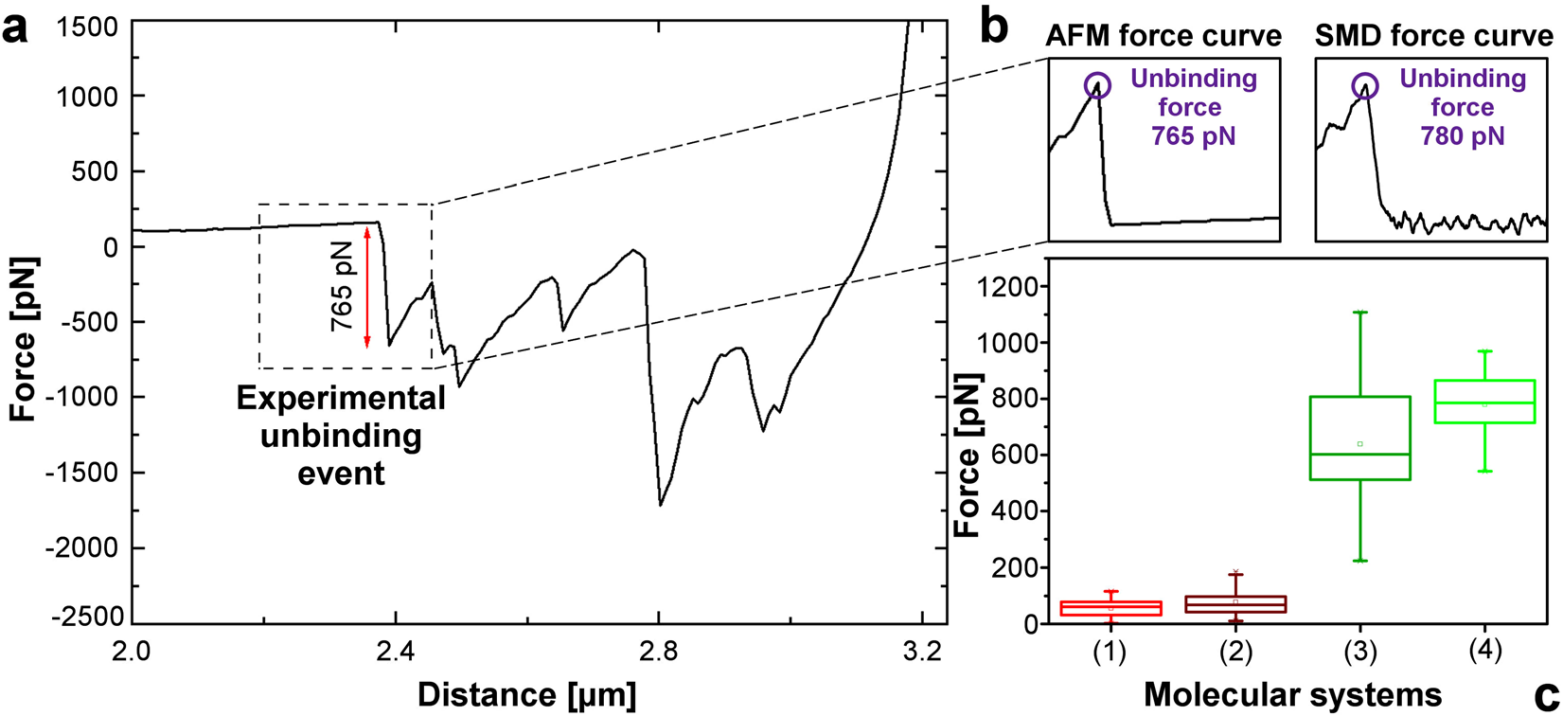
Interaction between MOG immunogenic peptide and antibody regarding force values. **(a)** AFM force curve of the complex formed by MOG_92–106_ and rabbit anti-MOG IgG. **(b)** Obtained AFM force curve in comparison with SMD data. **(c)** Boxplot of the AFM and SMD data including control measurements.

The obtained SMD force value for the MOG_92–106_-Fv portion detachment process is in agreement with the AFM measurements of the same complex, as shown in Fig. 5. AFM force curve (Fig. 5a) indicated an adhesion force (Fad) value of 765 pN, which is included in the AFM boxplot variation range represented in Fig. 5c. Fig. 5b presents the similarity between computational and AFM information, especially considering force values and curve shape. Systems in the Fig. 5c are described regarding the interaction between (1) unfunctionalised tip and rabbit anti-MOG; (2) peptide MOG_92–106_ and unspecific antibody (anti-glutathione-S-transferase); (3) peptide MOG_92–106_ and specific antibody (rabbit anti-MOG_92–106_); and (4) SMD data concerning MOG_92–106_ and anti-MOG binding.

**Figure 6:**
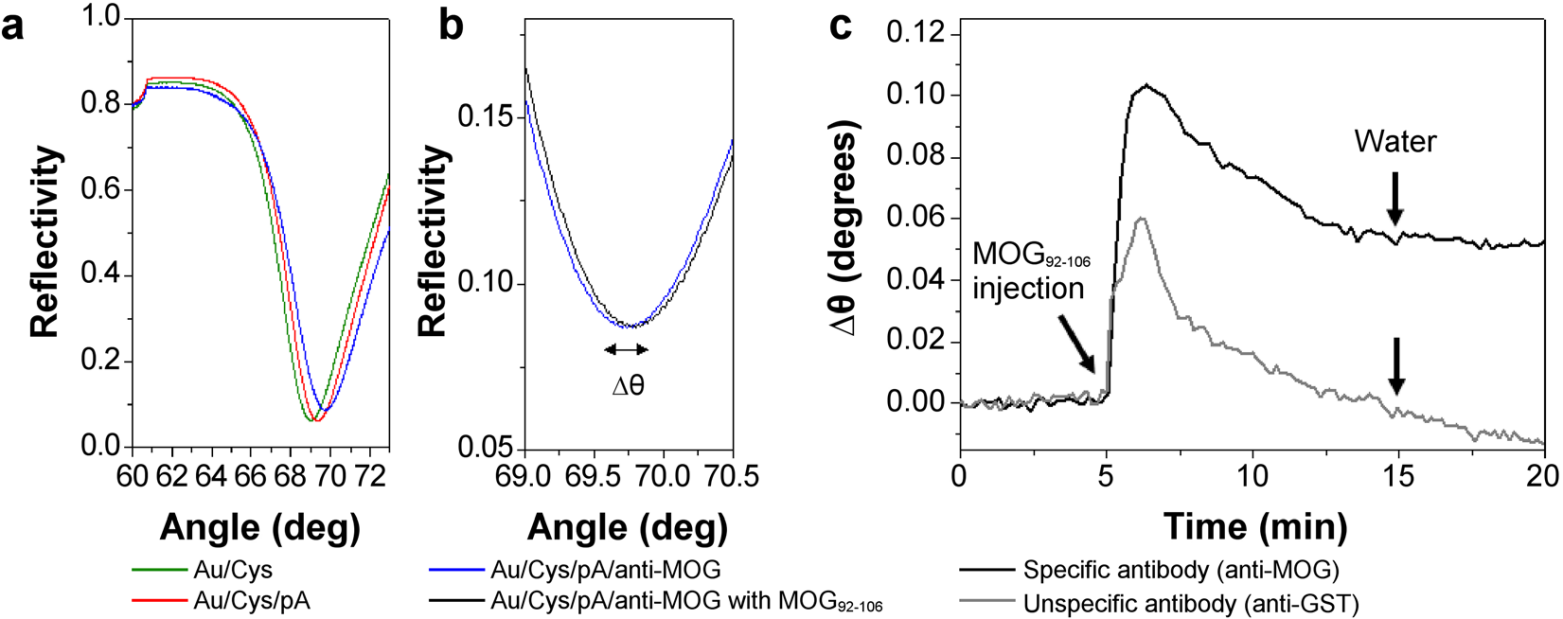
**(a)** SPR angular scan of the Au/Cys/pA/anti-MOG sensing surface assembly: Au/Cys in green, Au/Cys/pA in red, and Au/Cys/pA/anti-MOG (sensing surface) in blue; **(b)** SPR angle shift Δ*θ* due to the specific interaction between the sensing surface in blue with MOG_92–106_ peptide in black. **(c)** Real-time Δ*θ* monitoring during the MOG_92–106_ followed by water injection over the specific anti-MOG in black and unspecific anti-GST surface in grey.

Surface plasmon resonance (SPR) measurements were carried out in order to confirm the antigen-antibody binding (Fig. 6). The SPR analysis followed the tip functionalisation steps regarding layers deposition via injection. Fig. 6a depicts the angular scan corresponding to the gold sensor functionalised with a self-assembled monolayer of cysteamine hydrochloride (Au/Cys) followed by a protein A (Au/Cys/pA) and the anti-MOG layers (Au/Cys/pA/anti-MOG). A shift in the SPR angle of 0.077° was verified due to the specific interaction with MOG_92–106_ (Fig. 6b). To evidence the specificity of the interaction between anti-MOG with MOG_92–106_, an unspecific antibody (anti-GST) was also tested to the MOG_92–106_ injection. Fig. 6c depicts the real-time behaviour of the systems involving anti-MOG and anti-GST after the injection of MOG_92–106_.

## Discussion

This study involved a deep computational investigation of the MOG-antibody complex in aqueous solution. For a significant nanoscale description, available data on MOG structure and its specific antibody^6,21^ were taken into account together with considerations from the experimental-theoretical interface background of our research group. This study presented the following highlights: (1) Fab portion fluctuation of autoantibodies occurs during the antigen binding; (2) hydrogen bonds and salt bridges are important to the antigen-antibody complex structure maintenance, presenting values inversely related along the time; (3) CDR contribution is significant for properly antigen binding, especially regarding heavy chain residues; (4) electrostatics seems to be decisive during MOG recognition and binding by an antibody; and (5) the peptide MOG_92–106_ function in the binding process indicated its role as an anchor during MOG external domain recognition by demyelinating antibodies. Each finding is discussed with details in the following paragraphs.

Firstly, a distinct variation profile was noticed between anti-MOG Fab and MOG external domain (Fig. 1a), which showed to be strictly related to the molecule function in the organism. The higher flexibility degree of the anti-MOG Fab structure was expected since it comprises several loops and their mobility is required for a proper antigen binding^22,23^. Indeed, anti-MOG Fab fluctuation was more influenced by the variable than the constant region (Fig. 1b). Similarly, a more rigid MOG structure, presenting a less variation, is demanded since this protein seems to act in the CNS myelin structure maintenance^5^.

Hydrogen bonds and salt bridges were useful parameters for the system hydration effects, 3D structure maintenance, and structural stability analysis^24–26^. As can be seen from Table 1, hydrogen bonds and salt bridges values are inversely proportional, which is in agreement with Franca *et al*.^27^ and our previous work^28^ results. This situation may be explained by two factors: (i) salt bridges are weakened by solvation effects^26^, and (ii) charged amino acids induce new hydrogen bonds with water molecules^27,28^. Thus, the number of salt bridges decreases and, consequently, the conformational fluctuation is induced along the simulation (Fig. 1a). Additionally, low values of standard deviation of both hydrogen bonds and salt bridges revealed a small variation among measured values. Thus, structural stability and maintenance during the simulation are indicated.

Concerning antigen-antibody interaction, hydrogen bonds presented a pivotal role in complex formation and maintenance. CDR actively supported the antigen binding by the anti-MOG Fab portion, particularly CDR-H3 and H2 in the heavy chain. Light chain CDR, such as L1 and L2, presented a minor contribution in the antigen-antibody binding. Osajima and colleagues^29,30^ obtained similar results for hydrogen bonds during MD simulations of several Fab-antigen complexes, especially considering a CDR-H3 highest contribution and a CDR-L2 smallest contribution. The most interactive region of MOG was identified, which comprised thirteen residues that anchored the antibody binding. Ten of these residues are related to three MOG peptides referred as encephalitogenic in the literature: MOG_1–22_^31^ (GLY_1_, and GLN_4_), MOG_35–55_^13^ (TYR_40_, ARG_52_, and ASN_53_), and MOG_92–106_^31^ (ASP_102_, HIS_103_, SER_104_, TYR_105_, and GLN_106_). Also, we found three residues of MOG (THR_33_, GLU_107_, and GLU_108_) closely located to the three referred immunogenic peptides with a relevant contribution to the antigen-antibody complex.

Interestingly, Yannakakis and colleagues^16^ demonstrated key residues of MOG_35–55_ in the T-cells stimulation process during the interaction of Human Leukocyte Antigen (HLA), MOG_35–55_, and T-cell Receptor (TCR) using MD simulation. These results suggested the participation of some MOG residues, particularly TYR_40_ and ARG_52_, during the T-cell antigen presentation process. Thus, in comparison with our findings, a correlation between the processes of cellular and humoral response is evidenced with an overlap of key anchor residues.

The measured affinity involved in the antigen-antibody complex complemented our findings on hydrogen bonds established between MOG and anti-MOG Fab molecules. The obtained ΔG_bind_ average value (−43.1 ± 17.6 kcal mol^−1^) is in agreement with several Fab-antigen complexes analysed by Osajima *et al*.^29,30^, who calculated similar values of ΔG_bind_ for these complexes with a major electrostatic contribution for antigen-antibody binding. This finding was expected since electrostatic forces and energies play a central role in the specificity and interaction between biological macromolecules, especially proteins, which are highly charged (Fig. 3b)^32,33^. According to our data, electrostatic contributions were decisive for the affinity and highly specific antigen-antibody binding, which can be reflected regarding salt bridges and hydrogen bonds formed during the complex maintenance^33^.

Fig. 3c,d highlight the most interactive region of the MOG and anti-MOG Fab, respectively, with their charges reflected by electrostatic potential. These regions showed to be oppositely charged and, thus, presented a strong interaction during the simulations. They comprise both significant interactive CDR of Fab (H3, H2, and L3) and MOG residues strongly involved in hydrogen bonds (Fig. 2c). So, surface charges showed to be essential for the MOG and Fab binding.

Considering the information extracted from this MD simulation, we selected the MOG_92–106_ peptide for binding forces analysis by SMD and AFM methods. This peptide showed to actively participate in the interaction with the antibody since it involved a higher number of anchor residues. SMD data were obtained for MOG-Fv and MOG_92–106_-Fv interaction systems to precisely understand the contribution of specific residues for antigen-antibody binding. For the MOG-Fv system, the obtained results (Fig. 4a) complied with Su and Wang^34^ study, which presented comparable unbinding force values (~2,000 pN) and shape of SMD force curve for a similar system. For the MOG_92–106_-Fv system, computational data acquired were successfully validated by AFM measurements considering a similar antigen-antibody complex as shown in Fig. 5.

Fig. 5c boxplots highlight the force values variation of both AFM and SMD experiments, in which 100% of the obtained computational forces were included in the AFM force value range. The position of the SMD median value is contained in the AFM boxplot range, which comprises 50% of the representative force values of experimental adhesion events. This fact denotes the correspondence between the median values of SMD and AFM measurements. Also, these median values were distinct from those presented by control measurements, validating our data.

SPR data experimentally demonstrated both AFM tip functionalisation and antigen-antibody binding. Firstly, Fig. 6a,b evidence the assembling of the sensing surface which each curve shift indicates a new layer deposition. Secondly, binding and specificity of the complex composed of anti-MOG and MOG_92–106_ could be confirmed (Fig. 6c). In the anti-MOG system, the SPR shift remained after removing the MOG_92–106_ from the reaction channel via water flux. This occurred due to the specific interaction and formation of the antibody-peptide complex. In the anti-GST system, the SPR signal returned to initial values after removal of the MOG_92–106_ solution. This was expected considering the absence of interaction between MOG_92–106_ and anti-GST.

Fig. 4a compared with Fig. 4b suggested a significant contribution of the MOG_92–106_ peptide to the antigen-antibody interaction concerning forces involved in the complex maintenance. The force values computationally measured for both systems indicate that the force needed to detach the MOG_92–106_ peptide represent 75% of the total force to completely remove MOG external domain from the complex with the antibody. Thereby, our results suggest a decisive contribution of the MOG_92–106_ in the MOG recognition by specific demyelinating antibodies, highlighting this peptide as an epitope in the binding of the MOG external domain. Computer-aided techniques showed to be valuable tools in epitope characterisation^35,36^. In this study, we successfully employed MD and SMD approaches in the identification of the epitope recognized by MOG-demyelinating antibodies for the first time.

To sum up, the present study has provided detailed information concerning the antibody recognition of MOG, a relevant protein in demyelinating disorders. MD and SMD simulations successfully provided molecular details about MOG external domain and its specific antibody interaction. These analyses highlighted several residues related to three encephalitogenic peptides of MOG (MOG_1–22_, MOG_35–55_, and MOG_92–106_) with a significant contribution to the maintenance of the MOG-Fab complex. The affinity and specificity between MOG and the anti-MOG Fab portion were analysed and efficiently proved by computational, AFM, and SPR data. The SMD detachment force for the complex was successfully confirmed by AFM and suggests an important role for the MOG_92–106_ in the MOG recognition process, holding the entire complex. Further studies involving sample analysis of patients with demyelinating diseases using the sensor device described here could be interesting to confirm the computational results of this paper, as performed in similar studies in our research group^15,37–39^. Significant MOG descriptive data were generated and complied with several decades of MOG research, especially using animal models as EAE^11,13^. In this context, the application of combined methods as presented here can contribute to the investigation of new molecules related to autoimmune demyelinating disorders.

## Methods

### System preparation

The initial antigen-antibody 3D structure was obtained based on the crystallographic data of the interaction between the Fab portion of the demyelinating MOG-specific antibody 8–18C5 and MOG external domain from protein data bank (PDB) (PDB ID 1PKQ)^6^. The complex model included some modifications in the crystallographic structure by adding missing residues and hydrogen atoms according to Franca *et al*.^27^ protocol. Then, the refined complex model was inserted in a TIP3P^40^ simulation box. Some adjustments in the interaction system were assigned before the simulation. Firstly, the C*α* atom of the residue number 440 (CYS) in the heavy chain was fixed to mimic the presence of the IgG Fc (fragment crystallizable) portion. Secondly, considering the crystallographic data referring to the MOG extracellular domain description, the C*α* atom of the residue number 121 (PHE) in the MOG chain was also fixed, mimicking the presence of intact MOG protein in the myelin plasma membrane.

### Molecular Dynamics (MD) simulation and Free-binding energy calculation

MD simulation was carried out using CHARMM36 force field^41^ within the NAMD 2.9 program^42^. The input system was minimized under NVT ensemble condition, considering a time step of 2 fs, a cutoff distance of 12 Å for short-range interactions, and particle-mesh Ewald (PME) formalism^43^. The system temperature was gradually increased to 310 K, and then the equilibration step occurred during 200 ns under NpT ensemble condition at 1 atm and 310 K, respectively, using a Langevin piston^44^ and thermostat. MD trajectory analysis considered hydrogen bonds and salt bridges formation as well as RMSD of the biomolecules. Hydrogen bonds were quantified via Visual Molecular Dynamics (VMD)^45^ Hbonds plugin following computational protocols applied for similar antibody systems in the literature^29,30,46^. A hydrogen bond was quantified when the distance between a hydrogen donor (D) and an acceptor atom (A) was shorter than 3.5 Å as well as the angle H-D-A was shorter than 60.0°. Salt bridges formation was monitored via VMD Salt Bridge plugin according to Ierich *et al*.^28^ protocol. A salt bridge was quantified when the distance between the oxygen atom of an acidic residue and the nitrogen atom of a basic residue was shorter than 3.2 Å. Additionally, differences between the initial atomic coordinates and new positions assumed by residues during the simulation were measured using RMSD values.

Finally, the MOG-Fab complex free-binding energy (ΔG_bind_) was measured from parts of the 200 ns MD simulation trajectory with less structural fluctuation. A total of 15 ns (15,000 frames) was selected for ΔG_bind_ analysis. The estimation of ΔG_bind_ occurred based on the average of the binding energies values obtained for every 10 ps using molecular mechanics combined with the Poisson-Boltzmann surface area (MM/PBSA) method^47^. MM/PBSA was applied by Calculation of Free Energy (CaFE) plugin^48^ implemented to VMD program. Additional trajectories required for ΔGbind calculation were obtained using the same protocol described for antigen-antibody complex MD simulation. Poisson-Boltzmann calculation was carried out by APBS program.

### SMD simulation protocol

**Figure 7:**
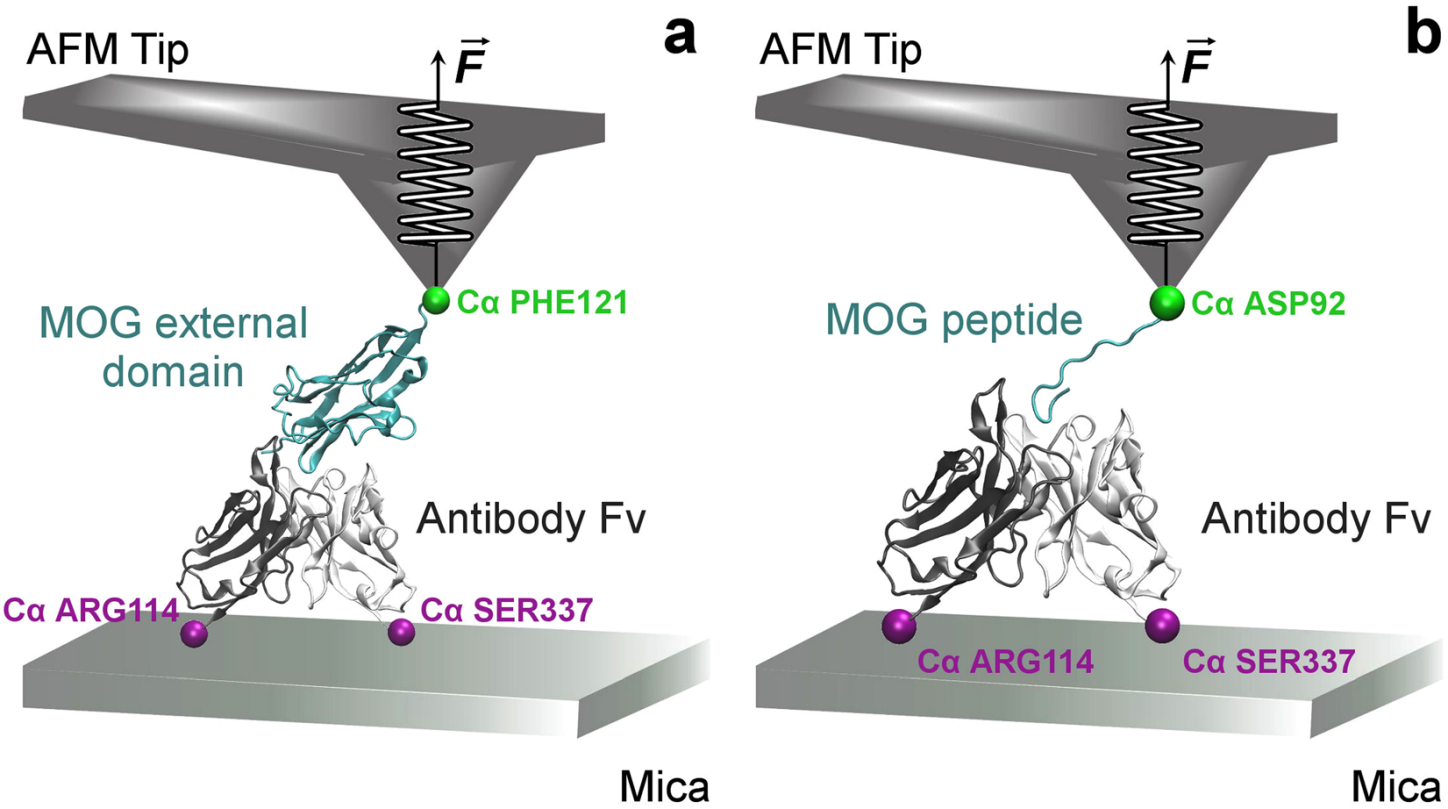
Schematic model of the SMD simulated systems with AFM tip and mica substrate indicated in representative positions. **(a)** SMD input of the complex MOG-Fv. **(b)** SMD input of the complex MOG_92–106_-Fv.

SMD simulations were performed considering the Fv portion of Fab in complex with (a) MOG external domain, and (b) MOG_92–106_ peptide (Fig. 7).

In the first system (Fig. 7a) the C*α* atoms of the residues ARG_114_ and SER_337_ were fixed in the antibody. The external force was applied in the C*α* atom of the residue PHE_121_ of MOG external domain structure. In the second system (Fig. 7b), fixed atoms were the same, and the external force was applied in the C*α* atom of the first residue (ASP) of the MOG_92–106_ peptide. For each system, 20 SMD simulations of 3 ns were conducted independently using a spring constant k of 2.15 kcal (mol Å^2^)^−1^ at a constant pulling velocity of 0.00005 Å timestep^−1^ (0.025 Å ps^−1^).

### Atomic force microscope (AFM) measurements

The experiments involving a prototype of AFM-based sensor were performed in a Veeco AFM, Nanoscope VTM model (Veeco Instruments Inc, Plainview, New York, USA), Multimode-VS system, with PicoForce package. For these experiments, silicon nitride AFM tips (DNP-10, Bruker Nano Inc, Billerica, Massachusetts, USA) with a spring constant of 0.03 N/m estimated by Thermo tune^TM^ (Veeco Instruments Inc, Plainview, New York, USA), and a nominal radius of 20 nm were used. Firstly, tips were sterilized by UV-ozone (Procleaner^TM^ Pro, Salt Lake City, Utah, EUA). After sterilization process, tip surface was chemically modified. The immobilisation of MOG_92–106_ peptides (Peptide and Chemistry Laboratory of IQ-USP, University of São Paulo, São Paulo, SP, Brazil) was carried out on a layer composed of (3-aminopropyl)triethoxysilane (APTES, 99%, Sigma-AldrichⓇ, St. Louis, Missouri, USA) and polyethylene glycol (PEG, Sigma-AldrichⓇ, St. Louis, Missouri, USA).

In a similar protocol, the surface of mica substrate (Mica muscovite, Ted Pella Inc., Redding, California, USA) previously cleaved was sterilized, and rabbit IgG anti-MOG_92–106_ molecules (Rheabiotech, Campinas, SP, Brazil) were immobilised on the sample surface using protein A protocol^49^. Control experiments were carried out as follow: (1) unfunctionalised tip interacting with substrate treated as described earlier and (2) tip functionalised with MOG peptides interacting with unspecific antibodies (a commercial anti-glutathione-S-transferase). Force-distance curves were obtained in triplicate via AFM in a fluid cell, carried out in phosphate buffered saline pH 7.4 (Sigma-AldrichⓇ, St. Louis, Missouri, USA), and the measured adhesion forces were analysed by Origin program (OriginLab, Northampton, Massachusetts, EUA).

### Surface Plasmon resonance (SPR) experiment

SPR measurements were performed with the SPR Navi 200 system (BioNavis, Finland) using Kretschmann configuration^50^ and wavelength *λ* = 670 nm. Gold sensors (50 nm-thick, BioNavis, Finland) were cleaned with a mixture of 5 H_2_O:1 NH_4_OH:1 H_2_O_2_ (v/v) for 10 min at 80 °C and washed extensively with ultrapure water. After, the gold sensors were functionalised overnight with 25 mM aqueous cysteamine hydrochloride. The sensor surface was assembled by further adsorption of protein A (50 *μ*g mL^−1^ in water) and the layer of anti-MOG (50 *μ*g mL^−1^ in water). The control measurements (unspecific antibody) were performed with the anti-GST (50 *μ*g mL^−1^ in water). All the experiments were carried out at 2 °C using water as a carrier under flux of 15 *μ*L min^−1^.

## Data Availability

All data generated or analysed during this study are included in this paper.
